# A Preliminary Investigation of the Reliability of Acoustic Parameters of Voice through Smartphone Recordings in Individuals with Dysphonia

**DOI:** 10.5195/ijt.2023.6572

**Published:** 2023-12-12

**Authors:** Meet Nakum, Namita Joshi

**Affiliations:** 1 Bharati Vidyapeeth School of Audiology and Speech Language Pathology, Pune, Maharashtra, India; 2 Sampark e-polyclinic, Pune, Maharashtra, India

**Keywords:** Acoustic measures, Smartphones, Telepractice, Telerehabilitation

## Abstract

Telepractice offers the opportunity to receive care at home without risk of exposure to healthcare acquired infections, especially during a pandemic. Hence, establishing the reliability of the diagnosis of dysphonia via a smartphone is fundamental to providing an alternative service delivery model. A total of 20 participants participated in the study. Recordings of sentence-based voice samples were done using a standardized microphone and the software used in labs and on smartphones. Comparisons were made of acoustic and perceptual voice in real-time and recorded samples speech in persons with typical vs pathological voice. Results revealed no significant differences perceptually between real-time voice and recorded voice in individuals with typical and pathological voices. In acoustic analysis, there was no significant difference in Fundamental frequency (F0) and Auditory Voice Quality Index (AVQI) between real-time voice and recorded voice in individuals with typical and pathological voice.

Human voice is produced by the complex vibration of the vocal cords and modified by resonatory filters. Because of the very high complexity of human voice, a comprehensive test battery is required to fully assess vocal function. These tests may include direct visualization methods such as laryngoscopy and stroboscopy. Indirect assessment of voice may include acoustic assessment, perceptual evaluation (e.g., Voice Handicap Index, Consensus Auditory Perceptual Evaluation of Voice, etc.) and aerodynamic assessment. The use of acoustic assessment has become increasingly widespread in recent years because it is easily available, low cost, easy to interpret, non-time consuming, and non-invasive (Buder, 2000). Institutional and hospital clinics routinely use acoustic evaluation as a part of their daily evaluation of voice. The parameters of fundamental frequency, intensity, jitter, shimmer, and harmonic-to-noise ratio are the most common acoustic parameters typically evaluated. However, in daily clinical practice in India, auditory perceptual evaluation of voice is still the predominate method used by experienced listeners to identify pathological voices.

Acoustic voice signal has very minimal cycle-to-cycle variability, though it does have small variability in amplitude and frequency (Kumar & Bhatt, 2010). Therefore, even small error or variability in frequency during recording can create significant errors in perturbation parameters. Hence, the best current method for determining voice quality is the perceptual evaluation of voice. However, the perceptual rating scales employed by speech-language pathologists may be less sensitive than instrumental assessment to the small changes in voice quality that happen with emergent behavioural voice issues.

The COVID-19 pandemic brought about methodological changes for voice intervention. Telepractice has since become essential for the clinical evaluation of voice (Castillo-Allendes et al., 2020) and may include voice recording of a client in audio or video format, vocal hygiene diaries, online voice evaluation of acoustic, instrumental aerodynamic, non-instrumental aerodynamic assessment and perceptual measures and tele-biofeedback of voice performance (Grillo, 2017). Use of tele-practice for voice rehabilitation has increased over the past ten years and will continue to increase because it is cost saving, easily accessible, and as effective as in-person treatment. Furthermore, there is increasing client demand for remote treatment (Grillo, 2019). However, the low computer literacy of rural populations in India poses a challenge for providing telepractice based treatment.

Sustained vowels are the most commonly used voice recording sample because they are easy to produce and less influenced by dialectal variation. However, they do not reflect an individual's variation in daily voice, as sustained vowels lack voiceless elements, and do not exhibit prosodic variations in amplitude, frequency, rate, or stress (Zraick & Wendel et al., 2005). Conversational speech samples, on the other hand, are more representative of the individual's typical speaking voice (ASHA, 2018).

The lowest frequency of the signal is indicated by the cepstrum peak amplitude in proportion to other periodic components or disturbances that are present. The smoothed cepstral peak prominence (CPPs) is one of the cepstral measures that is most frequently utilized in the objective evaluation of the typical and pathological voice. In acoustic evaluation of dysphonia, parameters such as Fundamental frequency, CPPS (Cepstral Peak Prominence Smoothed) and AVQI (Acoustic Voice Quality Index) have greater validity and reliability. They have the ability to assess voice quality even in a severely dysphonic voice (Heman-Ackah et al., 2002). These parameters also have less variability across different ages and genders. Previous research has also demonstrated that CPP has a high correlation with the categorization of auditory perception of dysphonia and most accurately predicts dysphonia in samples of connected speech (Maryn & Roy 2010). Obtaining a voice sample and carrying out acoustical analysis via synchronous or asynchronous telepractice is convenient for the clients who opt to have assessment through telepractice. Telepractice allows patients to receive care in their home without exposure to a healthcare acquired infection. Hence, establishing the reliability of telecommunication devices is fundamental to evaluating the efficacy of this alternative service delivery model.

The main aim of this study was to investigate the potential of identifying individuals with dysphonia via speech recorded on a smartphone (Android). A first step was to compare the acoustic parameters of individuals with and without dysphonia on recorded samples. A second step was to compare auditory perceptual scores and acoustic parameters scores of real-time voice versus recorded voice via telepractice for persons with non-pathologic vs. pathological voice.

## Method

### Participants

Twenty speakers, ages 20–35 years, participated in this study. None were receiving speech therapy. Ten had perceptually typical (i.e., non-pathologic) voices (5 males and 5 females) and ten had pathological vocal issues (5 males and 5 females). Two experienced speech-language pathologists rated the individuals on the GRBAS scale.

**Table 1.** Inclusion Criteria for Typical and Pathological Voice

Inclusion Criteria for Typical Voice	Inclusion Criteria for Pathological Voice
Individuals with a score of less than 1 in GRBAS with age-appropriate Body Mass Index (BMI) were included.	Individuals with a score between 1 & 2 in GRBAS included with age-appropriate Body Mass Index (BMI) were included.
Individuals with perceptually normal voice in terms of pitch, loudness, and intensity were included.	Individuals with perceptually mild dysphonic voice in terms of pitch, loudness, and intensity were included.
Participants who are not taking medication for hypertension, asthma, diabetes, gastro-oesophageal reflux disorders, or allergies were included.	Participants who have not undergone surgery related to voice were included.
Individuals who have not participated in voice rehabilitation in the past or present were included.	Individuals who have not participated in voice rehabilitation in the past or present were included.

Vocal health was determined by the subject as well as by the clinicians perceptually during conversation with the participants. All participants in the study provided written informed consent prior to enrolment. The informed consent form was approved by the Institutional Review Board and clearly explained the purpose of the study, the procedures involved, the risks and benefits of participation, and the right to withdraw from the study at any time.

### Procedure

GRBAS and the auditory perceptual rating scale were administered to perceptually evaluate the voice prior to the collection of actual voice samples. All the recordings were done in a voice lab with ambient noise less than 50dB measured via a sound level meter. The participants were asked to produce six sentences from the Consensus Auditory Perceptual Evaluation of Voice (CAPE – V). Speech productions were recorded by the researchers via the Audio Technica ATR2500 – USB Cardioid Condenser USB Microphone (Audio – Technica U.S., Inc.), used as a reference microphone. It is specifically designed for computer-based recording via any recording software. The microphone has a high-quality analog to digital converter with a 16-bit, 44.1/48 kHz sampling rate and is compatible with Windows and iOS devices. Its low mass diaphragm provides a frequency response from 30 to 15 kHz. The microphone was connected to a PC and positioned in front of the participant with a measured mouth-to-microphone distance of 15 cm or less. A table mounted stand was used to maintain the proper distance. Recording was done in PRAAT software (Version 6.1.16) with 44.1 kHz sampling rate and 32-bit resolution. All recordings were saved in .wav file. Each participant's sentence productions were analysed using the Praat software (Version 6.1.16) in the PC. At the same time, two graduate level trained speech-language pathologists rated the voice sample perceptually based on CAPE – V. Intra-class correlation coefficient was used to determine the inter-rater reliability between two raters with the same subjects. ICC varied from 0.66 for breathiness to 0.87 for loudness between rater 1 and rater 2 in real-time voice. There was moderate to good reliability of real-time voice between rater 1 and rater 2. ICC varied from 0.92 for loudness to 0.97 for overall severity between rater 1 and rater 2 in recorded voice, indicative of excellent reliability (ICC > 0.9) for recorded voice between rater 1 and rater 2.

A voice sample was next recorded by a smartphone (Android smartphone - which is most commonly used in India) in a natural environment setting (i.e., home environment) preferably at early morning or late night when the least ambient noise was present. Participants were instructed to switch off any fans and close the windows and doors while recording the voice sample. They were also instructed to maintain a microphone-to-mouth distance ≤ 4cm. This distance was pre-measured, and a mobile stand was used to maintain the distance. The voice samples were recorded via the Smart Voice Recorder app and parameters such as sample rate (i.e., 44.1 kHz) and microphone adjustment were kept constant during recording. An asynchronous method of telepractice was used, wherein each participant was asked to e-mail the voice sample recordings in a .wav format. The same recordings were sent to two SLPs without providing the demographic details for the rating based on CAPE – V or the Praat (Version 6.1.16) analysis by the researcher.

### Acoustic Analysis

The Cepstral Peak Prominence (CPPs) of all sample subjects were calculated in decibels (dB) using the Praat v. 6.1.16 program. The following configuration was used to calculate the CPPs. The audio recordings of the sample subjects were imported into Praat. The periodicity of the audio recordings was analyzed. A power cepstrogram was created from the audio recordings. The minimum pitch was set to 60 Hz. The time step was set to 0.002 seconds. The maximum frequency was set to 5000 Hz. Pre-emphasis was applied with a cut-off frequency of 50 Hz. The CPPs were calculated from the power cepstrogram. The CPPs were extracted from the power cepstrogram. Tilt was not subtracted before smoothing. The time averaging window was set to 0.01 seconds. The quefrency averaging window was set to 0.001 seconds. The peak search pitch range was set to 60 to 330 Hz. The tolerance was set to 0.05. Interpolation was performed using a parabolic function. The tilt line quefrency range was set to 0.001 to 0 seconds. The line type was set to straight. The fit method was set to robust. The acoustic voice quality of all voice samples was evaluated using the AVQI script in Praat (6.6.16), a phonetics software program.

## Results

This study aimed to explore perceptual comparison and acoustic comparison between real-time voice and tele-recorded voice. Descriptive results of the acoustic parameters and paired t-test were used to measure the reliability of acoustic parameters in individuals with typical and pathological voice.

Mean value of CPPS for typical real-time voice and tele-recorded voice was 7.95dB and 7.37dB respectively and mean value of CPPS for pathological real-time voice and tele-recorded voice was 6.18dB and 6.02dB respectively. In addition, mean value of AVQI for typical real-time voice and tele-recorded voice was 1.62 and 2.52 respectively and mean value of AVQI for pathological real-time voice and tele-recorded voice was 4.28 and 5.33 respectively. (Table 2)

**Table 2.** Descriptive Results of CPPS and ACQI for Participants with Typical and Pathological Voice

Group		CPPS (real-time)	CPPS (tele-recorded)	AVQI (real-time)	AVQI (tele-recorded)
Individuals with typical voice	Mean	7.95	7.37493	1.627	2.528
	Median	8.30	7.82	1.63	2.32
	Standard Deviation	0.65	0.89	0.79	0.84
Individuals with pathological voice	Mean	6.1868	6.0298	4.285	5.334
	Median	6.44	6.27	4.16	4.59
	Standard Deviation	1.49	1.40	1.82	2.25

In Table 3, significance values for all six parameters of CAPE V are given for rater 1 and rater 2. As per the Wilcoxon sign rank test, no significant difference was obtained between real-time voice and tele-recorded voice (p>0.05) for both raters across six parameters. Hence, we can conclude that there is no significant difference perceptually between real-time voice and tele-recorded voice in individuals with typical voice.

**Table 3.** Perceptual Comparison between Real-time and Tele-recorded Voice for Rater 1 and Rater 2 in Individual with Typical Voice with Test Statistics and Significance Values

(Rec vs. Real-time) RATER 1			(Rec vs. Real-time) RATER 2	
Typical voice			Typical voice	
Parameters	Test statistics (z)	p value	Test statistics (z)	p value
Overall Severity	6.00	0.65	0.00	0.15
Roughness	1.50	0.41	0.00	0.31
Breathiness	0.00	0.31	1.50	1.00
Strain	0.00	0.31	0.00	1.00
Pitch	0.00	1.00	0.00	1.00
Loudness	2.50	0.78	0.00	1.00

In the Table 4, significance values for all six parameters of CAPE V are given for rater 1 and rater 2. As we can see in Table 4, on the Wilcoxon sign rank test, no significant difference was obtained between real-time voice and tele-recorded voice (p>0.05) for both raters across six parameters. Hence, we can conclude that there is no significant difference perceptually between real-time voice and tele-recorded voice in pathological voice subjects.

**Table 4.** Perceptual Comparison between Real-time and Tele-recorded Voice for Rater 1 and Rater 2 in Individual with Pathological Voice with Test Statistics and P Values

(Rec vs. Real-time) RATER 1			(Rec vs. Real-time) RATER 2	
Pathological voice			Pathological voice	
Parameters	Test statistics (z)	p value	Test statistics (z)	p value
Overall Severity	5.00	0.67	0.00	1.00
Roughness	2.00	0.56	1.50	1.00
Breathiness	1.50	1.00	2.00	0.65
Strain	3.00	0.15	1.00	0.31
Pitch	3.00	0.15	0.00	1.00
Loudness	1.00	0.31	0.00	1.00

A comparison of acoustic parameters of voice (F0, Shimmer, HNR, CPPS, and AVQI) between real-time and tele-recorded voice for participants with typical and pathological voice was done using the Paired t test. (Table 5)

**Table 5.** Acoustic Comparison between Tele-recorded Voice and Real-time Voice for the Sample of Sentences in Individuals with Typical and Pathological Voice Paired t-test

Acoustic parameters	Participants with typical voice		Participants with pathological voice	
	Test statistics	p value	Test statistics	p value
FO	0.92	0.38	1.12	0.29
Shimmer	0.90	0.38	3.56	0.00^**^
HNR	5.27	0.00^**^	3.27	0.01^*^
CPPS	2.24	0.05	1.14	0.28
AVQI	3.60	0.00^**^	3.47	0.00^**^

*Note.* F0 = Fundamental frequency, HNR = Harmonic to noise ratio, CPPS = Cepstral peak prominence smoothed, AVQI = Acoustic voice quality index.

* p < 0.05,

** p<0.01

For individuals with typical and pathological voice, there was no significant difference (p>0.05) in F0 and CPPS between real-time and tele-recorded voice. On the other hand, there was significant difference (p<0.05) in HNR, Shimmer and AVQI between real-time and tele-recorded voice in individuals with typical voice and pathological voice.

## Discussion

A comparison between perceptual evaluation of real-time voice and tele-recorded voice was done for typical and pathological voices. No perceptual difference was found between real-time voice and tele-recorded voice.

Acoustic voice analysis is a reliable and accurate way to measure the acoustic properties of the voice, rendering it a valuable tool for clinical and research purposes. The acoustic parameters used to assess voice quality are all based on measurements of frequency and amplitude perturbations. Cepstrum-based measures are a more robust way to estimate the periodicity of harmonics in a signal because they are less sensitive to noise and other disturbances. Cepstrum analysis is a powerful tool for quantifying voice characteristics, but it is most effective when used to compare typical and dysphonic voices in connected speech. This is because connected speech is a more realistic and natural way to use the voice and it provides a better representation of how the voice is used in everyday life. Typical voices were found to have a stronger cepstral peak than breathy and hoarse voices. This is because typical voices have a well-defined harmonic structure, while breathy and hoarse voices have a poorly defined harmonic structure. People with the pathological condition had lower CPPS values than the control group. This could be because people with the pathological condition had a glottic chink, which is a gap in the vocal folds that can cause a flat harmonic structure. A Lower CPPS value indicates a poorer voice quality. This means that the voice is more breathy, hoarse, or rough. As can be seen in Table 5, there was no significant difference in F0 and CPPS between real-time and tele-recorded voice in individuals with typical and pathological voice, because parameters are more robust and less sensitive to environmental noise and other disturbances. Hence, the F0 and CPPS parameters are more reliable than Shimmer, HNR and AVQI, when voice is recorded through asynchronous method of tele-mode.

The Acoustic Voice Quality Index (AVQI) is a measure of voice quality that can be used to assess both sustained vowels and connected speech. The AVQI is a numerical score that ranges from 0 to 10, with 0 indicating no dysphonia and 10 indicating severe dysphonia. Parameters in AVQI include, CPPS, HNR, Shimmer and long-term average spectrum. AVQI has been found to differentiate the pathological voice from typical voice with 79% accuracy (Barsties et al., 2019). A study by Barsties et al. (2019) found that age and gender do not have a significant impact on the Acoustic Voice Quality Index (AVQI). There is evidence that the Acoustic Voice Quality Index (AVQI) can be used to track changes in voice quality before and after voice therapy. This suggests that AVQI is a valuable tool for clinicians who are evaluating the effectiveness of voice therapy (Hosokawa et al., 2017). A normal value for AVQI in healthy adults is 2.3 (SD: 0.8) (Faham et al., 2021). In the present study the mean AVQI value for healthy male and female subjects was within normal range for both real-time and tele-recorded voice. In contrast, the mean AVQI value for dysphonic male and female subjects fell in an abnormal range for both real-time and tele-recorded voice.

In the analysis of sentences there was a significant difference between real-time voice and tele-recorded voice, in Shimmer, HNR and AVQI (p<0.00) in individuals with typical and pathological voice, because these parameters are more sensitive to surrounding noise; environmental noise might have triggered the difference AVQI. The parameter of shimmer was significantly different (p<0.05) in pathological voice only, as the examiner could not control the severity of dysphonia while collecting samples (Table 5). Further studies should be conducted on a large number of typical as well as pathological voices.

In summary, acoustic analysis of voice is crucial in identifying the minute changes in the voice of clients with dysphonia. This study highlighted the reliability of acoustic parameters of tele-recorded voice. We concluded that CPPS is a more reliable acoustic parameter of voice than the AVQI. Therefore, the use of this tool should be encouraged for clinical populations to experience the benefits of telepractice in the diagnosis and treatment of voice. However, a cautious use of the acoustic evaluation through telepractice is warranted with further results gathered over the larger population of persons with dysphonia. The present study included only one smartphone and one app to measure specific voice samples. Data could be gathered on different types of smartphones across genders.
